# Prevalence and general characteristics of dementia: a nationwide population-based study of electronic health records in Türkiye

**DOI:** 10.55730/1300-0144.5833

**Published:** 2024-03-23

**Authors:** Merve GÜNER, Meltem KOCA, Serdar CEYLAN, Arzu OKYAR BAŞ, Ayşe DİKMEER, Mert EŞME, Osman ÇELİK, Mustafa Okan AYVALI, Murat ÇAĞLAYAN, M. Mahir ÜLGÜ, Gülnihal Gökçe ÜNAL, Cafer BALCI, Naim ATA, Mustafa CANKURTARAN, Burcu Balam DOĞU, Şuayip BİRİNCİ

**Affiliations:** 1 Division of Geriatric Medicine, Department of Internal Medicine, Faculty of Medicine, Hacettepe University, Ankara, Turkiye; 2 Division of Geriatric Medicine, Department of Internal Medicine, Etlik City Hospital, Ankara, Turkiye; 3 Division of Geriatric Medicine, Department of Internal Medicine, Konya City Hospital, Konya, Turkiye; 4 Division of Geriatric Medicine, Department of Internal Medicine, Mersin City Hospital, Mersin, Turkiye; 5 Ankara Provincial Health Directorate, Republic of Türkiye, Ankara, Turkiye; 6 General Directorate of Health Information System, Republic of Türkiye Ministry of Health, Ankara, Turkiye; 7 Department of Medical Biochemistry, Yıldırım Beyazıt Training and Research Hospital, Ankara Turkiye; 8 Deputy Minister, Republic of Türkiye Ministry of Health, Ankara, Turkiye

**Keywords:** Dementia, prevalence, geriatrics

## Abstract

**Background/aim:**

Türkiye is a country with an increasing life expectancy and an older adult population in parallel with the rest of the world. Several national small-scale studies were performed regarding the prevalence and characteristics of dementia in Türkiye, and the results of these studies differ from each other. We aimed to determine the prevalence of dementia in Türkiye to present the demographic characteristics, the frequency of use of health services, and the management of dementia.

**Materials and methods:**

Patients aged 65 years and over with a diagnosis of any type of dementia between January 1, 2019, and December 31, 2020, were retrospectively screened from the electronic health records of the Ministry of Health using ICD-10 codes.

**Results:**

In 2019, the total number of dementia cases identified in individuals aged 65 years and older was 247,727, of whom 150,529 (60.8%) were women. In 2020, the total number of dementia cases identified in this age group was 233,949, with 142,878 (61.1%) of these cases being women. The rate of patients admitted to the emergency department was 72.3% and 66.2% of all dementia patients in 2019 and 2020, respectively. In terms of the use of outpatient clinics, most patients with dementia were admitted to neurology (71.0% in 2019 and 62.4% in 2020). The geriatric medicine outpatient clinic was the least used by patients with dementia both in 2019 and 2020.

**Conclusion:**

The prevalence of patients living with dementia in Türkiye is lower than the global average. This suggests that most dementia cases are overlooked, highlighting the need to raise dementia awareness both in the community and among primary health care providers who frequently encounter older individuals. The study is significant in that it is the first to show the nationwide frequency of dementia in Türkiye.

## 1. Introduction

Dementia is a chronic and progressive clinical syndrome causing impairment in cognitive functions beyond age-related changes according to the definition by the World Health Organization (WHO)^1^. The key features of dementia are the cognitive impairment affecting the daily activities, social life, and occupation of the patient, due to the loss of previously acquired skills. There may be certain levels of mood and behavioral changes, psychiatric symptoms, and motor disorders accompanying impairments in cognitive functions [1,2]. With the expansion of the older population all over the world, the number of people living with dementia is also increasing. It is thought that 50 million people worldwide are diagnosed with dementia and 10 million new cases are added to this number each year^2^. The fact that dementia has become an important problem for the health policies of societies has increased the importance of identifying modifiable causes, as well as nonmodifiable causes. Approximately 40% of dementia cases are theoretically preventable [3]. The most common cause of dementia is Alzheimer’s disease, which is responsible for 60% to 70% of dementia cases. According to WHO, deaths due to Alzheimer’s disease and dementia have risen to the seventh leading cause of death globally^1^. Furthermore, mortality due to dementia doubled between 2000 and 2019. In addition, the total global cost of dementia in terms of health expenditures is estimated to be $1.3 trillion as of 2019^1^.

Türkiye is a country with an increasing life expectancy and older adult population, in parallel with the rest of the world. The population aged 65 and over in Türkiye has increased by 22.5% over the past five years, reaching 7,953,555 people in 2020. Meanwhile, the proportion of the older adult population in relation to the total population increased from 8.2% in 2015 to 9.5% in 2020^3^. In 2021, the population aged 65 and over reached 8,245,124, with the proportion of older adults increasing to 9.74%^4^. In separate studies conducted in İstanbul in 2008 and İzmir in 2006, the prevalence of Alzheimer’s disease was found to be 20% and 22.9%, respectively [4,5]. According to data published in 2008 by Hacettepe University Faculty of Medicine, Alzheimer’s disease was observed in 8.2% of 1255 patients over the age of 65 who applied to the geriatrics outpatient clinic [6]. In another study conducted in 2008 at Hacettepe University, the frequency of Alzheimer’s disease was 14.1% while that of vascular dementia was 3.8% [7]. In another study conducted in Ankara, the frequency of dementia was found to be 13.7% in 2010 [8]. In a recent study published in 2021 with 500 people in the Sivas region, the frequency of dementia was found to be 16.8%. It was also identified that the frequency of dementia was associated with older age, female sex, marital status, and educational status [9]. In another study conducted with a relatively small number of patients in Eskişehir in 2009, the overall prevalence of dementia was found to be 8.4%. Age-specific prevalences were 2.2% for those aged 55–59 years, 5.3% for those aged 60–64 years, and 30.4% for those aged 75 and older [10]. In another study conducted in 2014 with 555 geriatric female patients, the frequency of dementia was found to be 39.4% [11]. All these data are from national small-scale studies conducted at different timelines, and there is there is a lack of sufficient data specific to age groups. Additionally, the results of these studies differ from each other. Therefore, knowing the frequency and characteristics of national dementia stands out as a basic and priority step for the development of dementia management and national health policies.

In light of all this information, this study aims to determine the prevalence of dementia among individuals aged 65 and over in Türkiye, a developing country. It also seeks to present the demographic characteristics of patients with dementia, the frequency of use of health services, and management of this geriatric syndrome.

## 2. Material and methods

### 2.1. Study population and data collection

Patients aged 65 years and over with a diagnosis of any type of dementia between January 1, 2019, and December 31, 2020, were retrospectively screened using e-nabız. E-nabız is an information system designed by the Turkish Ministry of Health, and it has been used since 2015 by every healthcare facility in Türkiye. In 2019, 7,610,144 people and in 2020 7,979,559 people were registered in e-nabız. Anyone who applies to any health institution has a record on e-nabız. Therefore, individuals with a treatment report and/or at least one prescription for a dementia diagnosis were screened nationwide. The presence of dementia was identified based on the database with the International Classification of Diseases and Injuries-10 (ICD-10) diagnostic codes. Geriatric syndromes including malnutrition, osteoporosis, hip fracture history, incontinence, and depression were also determined by ICD-10 diagnostic codes (F00 and subdivisions, F01 and its subdivisions, F02 and its subdivisions, F03, G30 and its subdivisions, G31, F32-34 and their subdivisions, F38 and its subdivisions, F39, N31 and subdivisions, N39.3, N39.4, R32, M80–82 and their subdivisions, S72 and its subdivisions, E43, E44 and subdivisions, E64 and its subdivisions, Z93 and its subdivisions). The database was double-checked to avoid repetitive data. Epidemiological data, medical features and treatment choices, admission to healthcare facilities, and mortality status were obtained from the Turkish Ministry of Health database, e-nabız. This study was carried out with the permission of the Turkish Ministry of Health.

### 2.2. Statistical analysis

SPSS for Windows v.23.0 (IBM Corp., Armonk, NY) was used for the statistical analyses. Variables were examined using visual and analytical methods to determine whether they were normally distributed. Categorical variables were shown as numbers and frequencies. Continuous data that followed a normal distribution were described with mean ± standard deviation (SD). When distributions were not normal, the data were described with median [Quartile 1–Quartile 3]. A 5% type I error level was used to infer statistical significance.

## 3. Results

The study population is the entire Turkish population aged ≥65 years with a diagnosis of dementia in two respective years, 2019 and 2020. From January 1, 2019, to December 31, 2019, the total number of dementia cases identified in individuals aged 65 years and older was 247,727, of whom 150,529 (60.8%) were women (Table 1). Between January 1, 2020, and December 31, 2020, the total number of dementia cases identified in individuals aged 65 years and older was 233,949, of whom 142,878 (61.1%) were women. The prevalence of dementia, which was presented per thousand, was 33‰ in 2019, whereas it was 29‰ in 2020. The median age of patients with dementia in the geriatric population was 79.0 [73–85], in 2019 and 80.0 [74–86] in 2020. The frequency of dementia was 100.0‰ among patients aged 85 years and older in both 2019 and 2020. For individuals aged 75–84 years, the frequency of dementia was 49‰ in 2019 and 45‰ in 2020. In the 65–74 age group, the frequency of dementia was 16‰ in 2019 and 13‰ in 2020. Table 1 shows the prevalence of dementia in the geriatric population in Türkiye by year.

The median number of use of healthcare facilities in patients with dementia was 40 [27–59] in 2019 and it was 31 [20–46] in 2020. The rate of patients admitted to the emergency department was 72.3% in 2019 and 66.2% in 2020 of all dementia patients. In 2019, 15,720 (6.3%) patients diagnosed with dementia were hospitalized in the intensive care unit (ICU) and 142,041 (57.3%) patients were hospitalized in other wards. The median length of stay in the hospital was 13 [6–28] days in 2019. In addition, in 2020, 8.0% of all dementia patients were hospitalized in ICU and 49.2% of the whole dementia population was hospitalized in other wards. The median length of hospital stay was 10 [4–20] days in 2020. The rate of patients with dementia followed by homecare services was 5.5% in 2019 and 7.5% in 2020. Regarding the use of outpatient clinics, it was observed that most of the patients with dementia (71.0% in 2019 and 62.4% in 2020) were admitted to neurology. The outpatient clinic of geriatric medicine was the least used by patients with dementia both in 2019 and 2020. The use of healthcare facilities in patients diagnosed with dementia in the geriatric population in Türkiye is shown in Table 2.

The most frequently observed chronic condition in patients with dementia was hypertension followed by atherosclerotic heart disease, diabetes mellitus, and cerebrovascular diseases, respectively (Table 3). Regarding geriatric syndromes, depression was the most commonly encountered condition in patients living with dementia in both years (54.4% and 53.7%, respectively). In 2019, 13.6% of the entire dementia population had osteoporosis while 2.2% experienced femur fractures. In 2020 9.5% of the patients living with dementia had osteoporosis while 2.1% experienced femur fractures. The malnutrition rate was 5.9% in 2019 and 7.3% in 2020. The frequency of urinary incontinence was 7.7% in 2019 and 5.6% in 2020 (Table 3).

The mostly prescribed antidementia medication was donepezil. The donepezil prescription rate was 16.6% in 2019 and 9.4% in 2020. N-methyl-D-aspartate (NMDA) antagonist, memantine, was the second most prescribed medication for dementia. In 2019, 9.3% of all dementia patients were prescribed memantine, while in 2020, 4.4% of patients with dementia were treated with this medication. The use of combination therapy rates was 7.3% and 3.2% in 2019 and 2020, respectively. While the use typical antipsychotic rate was 5.8% (n = 14,438) in 2019, it was 6.5% (n = 15,329) in 2020. The most commonly used first-generation antipsychotic was haloperidol (5.1% in 2019 and 5.9% in 2020). The most frequently used second-generation antipsychotics were quetiapine (18.5% in 2019 and 22.0% in 2020), olanzapine (4.9% in 2019 and 5.7% in 2020), and risperidone (2.6% in 2019 and 2.6% in 2020), respectively. Treatment choices for osteoporosis were bisphosphonates, denosumab, and teriparatide, respectively, in both 2019 and 2020. Antibiotics were prescribed to 60.4% of all dementia patients in 2019 and 49.6% in 2020. The use of analgesics was 53.9% in 2019 and 48.1% in 2020. Prescription-based medication use in patients diagnosed with dementia in the geriatric population in Türkiye is summarized in Table 4.

## 4. Discussion

As in the rest of the world, the population aged 65 years and over is increasing in our country, leading to a rise in the frequency of dementia as the geriatric population grows. Our study is essential since it is the first to reveal the prevalence of dementia diagnoses in Türkiye over the past two years. It has been observed that in our developing country, the number of dementia diagnoses is lower than the global prevalence, suggesting that many cases are overlooked. This highlights the need to raise awareness about dementia both in the community and among primary health care providers who frequently encounter older individuals. The study is of great importance in that it is the first study to present the nationwide prevalence of dementia in Türkiye.

A relative decrease in the frequency of dementia was observed in 2020, which can be attributed to the decrease in elective hospital admissions due to the Coronavirus Disease 2019 (COVID-19) pandemic. The fact that older adults experience the most severe course of COVID-19, with higher mortality rates, accounts for the restrictions introduced to protect them from the negative health consequences of the pandemic.

Dementia cases were more commonly observed in women in Türkiye, as is the case globally. Factors contributing to the higher frequency of dementia in women include their longer life expectancy and lower levels of education in Türkiye.

Studies have shown that the utilization of healthcare services by patients with dementia is largely due to increased hospitalizations. Other research indicates that patients with dementia experience more hospital admissions and aggressive interventions near the end of life compared to those without dementia. It has been suggested that patients with dementia have a higher frequency of hospitalizations and emergency department visits than those without the condition. A nationwide study from Taiwan investigating the healthcare utilization of patients with dementia revealed that 84.9% of patients with dementia were admitted to emergency services and 83.6% of patients with dementia were hospitalized. The median length of stay was 8.6 days per admission [12]. A retrospective cohort study from the USA reports that 78.5% of all hospitalizations of patients with dementia involved emergency department care. The mean length of stay in the hospital ranged from 5.9 to 6.1 days between 2012 and 2016. Additionally, 84.1% of emergency department hospitalizations and 4.4% of elective hospitalizations were caused by preventable conditions [13]. The emergency department was the most frequently utilized healthcare service by patients with dementia in Türkiye. In 2019, 72.3% of people with dementia visited the emergency department, and in 2020, 66.2% of people with dementia sought emergency care. Another striking finding of our study was the low rates of antidementia drug use and treatment compliance among the patients. Several factors may contribute to this low medication use, including the limited efficacy of antidementia medications, discontinuation of medications due to adverse effects such as gastrointestinal side effects, challenges with treatment adherence, and issues related to polypharmacy and inappropriate drug use in older people.

Geriatric syndromes, such as malnutrition, depression, and urinary incontinence, are frequently associated with dementia. A study conducted at a university hospital in Türkiye involving 253 patients with Alzheimer’s disease found that 25.3% had malnutrition, and 39.5% were at risk of malnutrition [14]. However, in our current study, the malnutrition rates were 5.9% in 2019 and 7.3% in 2020, which are notably lower than the previous findings. This discrepancy suggests that malnutrition may be overlooked and highlights the importance of comprehensive geriatric evaluations for patients with dementia. Yet, in Türkiye, only one in ten patients with dementia was evaluated by a geriatrician. Physicians’ awareness and ability to address geriatric syndromes may differ, impacting their likelihood of recognizing and documenting these conditions in clinical practice. Furthermore, differences in documentation practices among healthcare facilities can also influence the completeness and accuracy of medical records. Inconsistent documentation of symptoms or conditions related to malnutrition or other geriatric syndromes may result in these issues being overlooked during data collection and analysis.

Another common problem in patients with dementia is urinary incontinence. It is estimated that nearly half of the patients with dementia experience incontinence according to a study from the United Kingdom [15]. A systematic review reported that the prevalence of incontinence among patients with dementia ranges from 11% to 90%. It is a more commonly encountered problem in nursing homes, with a prevalence of urinary incontinence of 74% in such settings, compared to 32% among community-dwelling older adults with dementia [16]. On the other hand, in Türkiye, only 5.6%–7.7% of all patients with dementia had urinary incontinence, which is less than expected. The authors thought that the unregistered use of absorbent products for incontinence could be the reason for the low incidence. Another neglected problem was osteoporosis in accordance with our results. Osteoporosis often cooccurs with dementia, as both disorders are strongly related to old age and dementia has been associated with an increased risk of falls and hip fractures. Thus, dementia patients are at increased risk of fracture and potentially at increased risk of mortality should they sustain a fracture [17]. In a study from Sweden conducted on 305 dementia patients, any type of osteoporotic fracture was seen in 25.4% and hip fracture was found in 16% of patients with dementia, which are higher than our results. In another previous study, 5.4 % of patients were treated with osteoporosis drugs, but none of them received bisphosphonates [18]. In contrast, bisphosphonates were the drug of choice for osteoporosis in Türkiye.

Depression, in addition to being a risk factor and prodrome of dementia, is another geriatric syndrome frequently associated with dementia. Distinguishing depression from cognitive impairment can be challenging, and recognizing depression in individuals with dementia is not always straightforward [19]. The prevalence of depression in dementia varies across studies due to differences in diagnostic criteria and study populations; however, it is estimated that nearly 30% of patients with dementia also had depression [20]. In our study, the rate of depression was found to be higher than in previous studies.

A geriatric assessment aids in diagnosing medical conditions, developing treatment and follow-up plans, coordinating of management, and evaluating long-term care needs and optimal placement. Unlike a standard medical evaluation, a geriatric assessment includes non-medical domains, emphasizes functional capacity and quality of life, and often involves a multidisciplinary team. It typically provides a more complete and relevant list of medical, functional, and psychosocial problems [21].

Cholinesterase inhibitors have a minor effect on enhancing cognition and activities of daily living in patients with mild-to-moderate Alzheimer’s disease [22]. They are also indicated for severe Alzheimer’s disease. Memantine can be used either in combination with cholinesterase inhibitors or independently for patients with moderate to severe Alzheimer’s disease [23]. In our study, the rate of patients treated with anti-dementia medications was lower than expected. This decline is thought to be related to the COVID-19 pandemic. According to a decision by the Ministry of Health of the Republic of Türkiye, medications were made available without a prescription to prevent interruptions in patient treatment during the pandemic. The lower observed rates of anti-dementia treatments may be due to medications not being reflected in prescriptions and remaining unregistered.

Pain is a prevalent symptom affecting 32%–64% of community-dwelling people living with dementia. They often experience acute and chronic pain from various causes, including neuropathic or nociceptive pain. A study from Türkiye revealed that 48.7% of patients with early-stage dementia, 22.2% with moderate-stage dementia, and 27.3% with severe-stage dementia reported experiencing pain [1]. Regardless of the cause of pain, it requires thorough assessment and management to ensure the appropriate type and dosage of analgesia is provided. It was revealed that analgesics were used by 34.9% of persons with dementia according to a study conducted on 67,215 participants with dementia [24]. In contrast, another study stated that people with dementia had a consistently lower prevalence and odds of analgesic prescription compared with people without dementia [25]. In line with our results, one in every two patients was treated with analgesics; unfortunately, the type of analgesics was unknown. Recent reports from observational studies have indicated that the overall use of analgesics in people with dementia is increasing, most markedly for opioid analgesics; however, paracetamol is the first-line and the safest treatment for pain in patients with dementia [26].

Antipsychotic medicines are commonly used to treat behavioral and psychiatric symptoms of dementia. The American Psychiatric Association recommends the use of antipsychotics in patients with dementia in cases of dangerous agitation and psychosis. These medications can minimize the risk of violence, reduce patient distress, improve the patient’s quality of life, and reduce the burden on caregivers [27]. According to a metaepidemiological study, conventional antipsychotics had a small but insignificant effect on agitation in dementia while they showed a small treatment effect on psychosis in patients with dementia. Atypical antipsychotics had a minimal but statistically insignificant effect on agitation and a negligible statistically significant effect on psychosis [28]. A nationwide study from Denmark reported that antipsychotics increased the risk of mortality, so they should be prescribed with caution [29]. Mueller et al. revealed that only 11.0% of the whole study population had been prescribed any type of antipsychotics, and the use of antipsychotics was associated with increased stroke and mortality risk [30]. FDA published a black-box warning about antipsychotics since they are associated with increased rates of stroke and death in older adults with dementia [1]. According to our nationwide data, atypical antipsychotics were preferred over typical antipsychotics, with nearly one in every three patients with dementia being treated with atypical antipsychotics. However, our study did not include information on the indications, effects, or adverse events related to antipsychotic treatment.

Infections are common in people with dementia, and the use of antibiotics is widespread, though practices vary across healthcare settings and countries. The most frequently observed infections in patients with dementia include respiratory tract infections, urinary tract infections, and skin and soft tissue infections. Due to the atypical presentation of infections and the limited communication of symptoms as cognitive function declines, these individuals experience delays infection recognition and an increased risk of mortality, both overall and from infections. According to a narrative review, the rate of antibiotic use was reported to be 52% [31]. Similarly, despite variations in antibiotic prescription practices between countries and severity levels, approximately half of the patients with dementia in Türkiye were treated with antibiotics.

There were some limitations of the present study. Since data for the study were drawn using the ICD-10 diagnostic codes, the type and stage of dementia could not be obtained. Another important limitation is the lack of data on the causes of hospitalizations and deaths. Furthermore, we acknowledge that while our study provides valuable insights into the characteristic features of dementia patients, it may not delve deeply into the nuances surrounding the pathogenesis, diagnosis, and prognosis of the disease, which is another limitation. However, our primary aim was to highlight the demographic characteristics and trends within Türkiye’s older population. This study is of great importance as it is the first to evaluate dementia data on a nationwide scale.

In conclusion, it is striking that geriatric syndromes that increase mortality and morbidities, such as malnutrition, urinary incontinence, and osteoporosis, are often overlooked. Since dementia has begun to be evaluated as a public health problem by the World Health Organization, it is crucial to raise awareness of this issue, especially among primary care and emergency physicians. Comprehensive assessments by geriatricians can improve the quality of life for patients and their caregivers, and reduce morbidity and mortality. Referring patients with dementia to geriatricians and homecare services as needed is essential.

## Figures and Tables

**Table 1:** Prevalence of dementia in the geriatric population in Türkiye by year.

	2019		2020	
	n (%)	Prevalence	n (%)	Prevalence
**Total**	247,727 (100)	33‰	233,949 (100)	29‰
**Sex**				
**Female**	150,529 (60.8)	36‰	142,878 (61.1)	32‰
**Male**	97,198 (39.2)	29‰	91,071 (38.9)	26‰
**Age groups**				
**65–74 years**	73,749 (29.8)	16‰	66,146 (28.3)	13‰
**75–84 years**	105,482 (42.6)	49‰	100,949 (43.2)	45‰
**≥85 years**	68,496 (27.6)	100‰	66,854 (28.6)	100‰

The prevalence of dementia was presented per thousand.

**Table 2:** Use of healthcare facilities by patients diagnosed with dementia in the geriatric population of Türkiye.

	2019	2020
**Use of healthcare facilities per patient**	40 [27–59]	31 [20–46]
**Patients admitted to the emergency department**	179,134 (72.3)	154,853 (66.2)
**Patients hospitalized in ICU**	15,720 (6.3)	18,689 (8.0)
**Length of stay in ICU (days)**	5 [2–9]	5 [2–10]
**Patients hospitalized in other wards**	142,041 (57.3)	115,092 (49.2)
**Length of stay in hospital (days)**	13 [6–28]	10 [4–20]
**Patients followed by homecare services**	13,703 (5.5)	17,553 (7.5)
**Patients admitted to internal medicine clinics**	165,546 (66.8)	119,407 (51.0)
**Patients admitted to geriatric medicine clinics**	4129 (1.7)	3294 (1.4)
**Patients admitted to neurology clinics**	175,769 (71.0)	145,944 (62.4)
**Patients admitted to psychiatry clinics**	86,972 (35.1)	67,402 (28.8)
**Deceased**	101,959 (41.2)	84,385 (36.1)

* Variables given as n(%) or median [25^th^–75^th^].

**Table 3:** Chronic diseases, geriatric syndromes, and mortality in the geriatric population diagnosed with dementia in Türkiye.

	2019	2020
*Chronic Diseases*	n (%)	n (%)
**Hypertension**	195,154 (78.8)	174,013 (74.4)
**Atherosclerotic heart disease**	92,222 (37.2)	71,873 (30.7)
**Diabetes mellitus**	83,754 (33.8)	72,084 (30.8)
**Cerebrovascular disease**	75,654 (30.5)	64,192 (27.4)
**Hyperlipidemia**	54,364 (21.9)	40,604 (17.4)
**Chronic obstructive pulmonary disease/asthma**	73,311 (29.6)	56,325 (24.1)
**Malignancy**	14,846 (6.0)	12,678 (5.4)
Nonhematological	13,831 (5.6)	11,763 (5.0)
Hematological	1317 (0.5)	1159 (0.5)
**Atrial fibrillation**	31,366 (12.7)	25,923 (11.1)
**Chronic heart failure**	25,453 (10.3)	20,661 (8.8)
**Chronic renal disease**	16,156 (6.5)	13,474 (5.8)
***Geriatric Syndromes***		
Malnutrition	14,724 (5.9)	17,100 (7.3)
Depression	134,772 (54.4)	125,527 (53.7)
Osteoporosis	33,811 (13.6)	22,213 (9.5)
Hip fractures	5543 (2.2)	4900 (2.1)
Urinary incontinence	18,997 (7.7)	13,059 (5.6)

**Table 4:** Prescription-based medication use in patients diagnosed with dementia in the geriatric population of Türkiye.

	2019 n (%)	2020 n (%)
**Antidementia Drugs**		
Donepezil	41,109 (16.6)	22,006 (9.4)
Rivastigmine	16,214 (6.5)	7200 (3.1)
Galantamine	14 (0.01)	9 (0.005)
Memantine	23,161 (9.3)	10,323 (4.4)
Combination of donepezil and memantine	18,054 (7.3)	7515 (3.2)
Gingko biloba	31,727 (12.8)	16,094 (6.9)
**Antipsychotic Drugs**		
**Typical**	14,438 (5.8)	15,329 (6.5)
Haloperidol	12,594 (5.1)	13,874 (5.9)
**Atypical**	71,522 (28.9)	76,535 (32.7)
Quetiapine	45,855 (18.5)	51,356 (22.0)
Olanzapine	12,168 (4.9)	13,349 (5.7)
Risperidone	6368 (2.6)	6075 (2.6)
Aripiprazole	4705 (1.9)	3756 (1.6)
**Osteoporosis Treatment**		
Bisphosphonates	16,320 (6.6)	9034 (3.9)
Denosumab	2690 (1.1)	1600 (0.7)
Teriparatide	172 (0.1)	102 (0.1)
**Antibiotics**	149,702 (60.4)	116,115 (49.6)
**Analgesics**	133,418 (53.9)	112,543 (48.1)

## Footnotes

1 World Health Organization. Dementia [online] Website https://www.who.int/news-room/fact-sheets/detail/dementia. [Accessed 23 October 2022]

2 World Health Organization. Dementia, Prevalence [online] Website https://www.who.int/health-topics/dementia#tab=tab_2 [Accessed 23 October 2022]

3 Türkiye İstatistik Kurumu. İstatistiklerle Yaşlılar, 2020 [online] Website https://data.tuik.gov.tr/Bulten/Index?p=Istatistiklerle-Yaslilar-2020-37227 [Accessed 23 May 2022]

4 Türkiye İstatistik Kurumu. İstatistiklerle Yaşlılar, 2021 [online] Website https://data.tuik.gov.tr/Bulten/Index?p=Istatistiklerle-Yaslilar-2021-45636 [Accessed 23 May 2022]
